# Retraction: *Syzygium aqueum*: A polyphenol- rich leaf extract exhibits antioxidant, hepatoprotective, pain-killing and anti-inflammatory activities in animal models

**DOI:** 10.3389/fphar.2024.1515827

**Published:** 2024-10-29

**Authors:** Frontiers Editorial Office

The Journal retracts the 5 June 2018 article cited above.

Following publication, concerns were raised regarding the integrity of the images in the published figures. Image duplication was identified in **Figure 5**. The image in this article also appeared in two other articles with common authors which described the rats as Male Sprague-Dawley rats [in (Frontiers in Pharmacology (2018), doi: 10.3389/fphar.2018.00566) and (Food and Chemical Toxicology (2018), doi: 10.1016/j.fct.2018.01.031]) and as Male Wistar rats [in (Pharmaceuticals (2020), doi: 10.3390/ph13050084)]. The authors failed to provide a satisfactory explanation during the investigation, which was conducted in accordance with Frontiers’ policies. As a result, the data and conclusions of the article have been deemed unreliable and the article has been retracted.

This retraction was approved by the Chief Editors of Frontiers in Pharmacology and the Chief Executive Editor of Frontiers. The authors did not agree to this retraction.

